# Identification of key genes in coronary artery disease: an integrative approach based on weighted gene co-expression network analysis and their correlation with immune infiltration

**DOI:** 10.18632/aging.202638

**Published:** 2021-03-03

**Authors:** Yang Yang, Xiangshan Xu

**Affiliations:** 1 Fourth Affiliated Hospital of China Medical University, Huanggu, Shenyang 110032, Liaoning, China

**Keywords:** weighted gene co-expression network analysis, least absolute shrinkage and selection operator, immune cell infiltration, coronary artery disease

## Abstract

This study aimed to identify key genes related to coronary artery disease (CAD) and its association with immune cells infiltration. GSE20680 and GSE20681 were downloaded from GEO. We identified red and pink modules in WGCNA analysis and found 104 genes in these two modules. Next, least absolute shrinkage and selection operator (LASSO) logistic regression was used to screen and verify the diagnostic markers of CAD. We identified ASCC2, LRRC18, and SLC25A37 as the key genes in CAD diagnosis. We further studied the immune cells infiltration in CAD patients with CIBERSORT, and the correlation between key genes and infiltrating immune cells was analyzed. We also found immune cells, including macrophages M0, mast cells resting and T cells CD8, were associated with ASCC2, LRRC18 and SLC25A37. Gene enrichment analysis indicated that these genes mainly enriched in apoptotic signaling pathway for biological pathway analysis, riboflavin metabolism for KEGG analysis. The diagnostic efficiency of these key genes measured by AUC in the training set, testing set and validation cohort was 0.92, 0.96 and 0.83, respectively. In conclusion, ASCC2, LRRC18 and SLC25A37 can be used as diagnostic markers of CAD, and immune cell infiltration plays an important role in the onset and development of CAD.

## INTRODUCTION

Atherosclerosis is the major cause of cardiovascular disease all over the world and brings heavy burden to the society, especially in China [1]. To accurately diagnose coronary artery disease (CAD), coronary artery contrast CT and cardio-angiography are widely used in China and they display high diagnostic accuracy [2]. However, these diagnostic methods need specialized medical centers and experienced cardiologists, which restrict its routine use in clinical practice. Recently, circulating mRNA expression in peripheral blood has been found to be correlated with tumor [3], hypertension [4] and diabetes [5]. However, the diagnostic value of mRNA in peripheral blood sample in CAD patients remains unclear.

Weighted Gene Co-expression Network Analysis (WGCNA) is an advanced systems biology-based approach used for finding molecular mechanisms and for linking the information to phenotypic traits [6]. WGCNA has been widely and successfully used to identify candidate biomarkers and therapeutic targets in various diseases. For example, by using WGCNA analytic method, Giulietti [7] revealed key genes involved in pancreatic ductal adenocarcinoma development. Recently, machine learning significantly improved the accuracy and predictive value of the computational models based on key genes identified from microarray or next generation sequencing data [8]. Least absolute shrinkage and selection operator (LASSO), a widely used machine learning method to prevent overfitting by using L1 regularization, was used to identify the hub genes [9]. However, there are few studies which have combined using WGCNA and LASSO to identify key genes in CAD.

Recently, accumulating studies indicated that immune cell infiltration may have an important effect on the onset and progression of CAD. Therefore, it is of great importance to assess the immune cells infiltration and evaluate the different types of infiltrating immune cells to clarify the molecular mechanism underlying CAD [10]. CIBERSORT is a widely used analysis tool using microarray data or RNA-seq data to investigate the expression profile of 22 types of immune cells and to calculate the proportions of each type of immune cells in the samples [11]. However, as far as I know, CIBERSORT has not yet been used to analyze the infiltration of immune cells in CAD.

In present study, we downloaded the gene expression profile of CAD and control patients from Gene Expression Omnibus (GEO) and the top 25% genes with high expression variance were analyzed by WGCNA method. Key modules closely associated with CAD were identified. The potential function of genes in these key modules was analyzed by Gene Ontology (GO) and Kyoto Encyclopedia of Genes and Genomes (KEGG). Key genes were identified by LASSO method from the genes in key modules. Then, the diagnostic efficiency of key genes was further validated in training set, testing set and validation cohort. Besides, we evaluated the association between key genes and immune cell infiltration.

## RESULTS

### Batch effect removal

**Figure 1:**
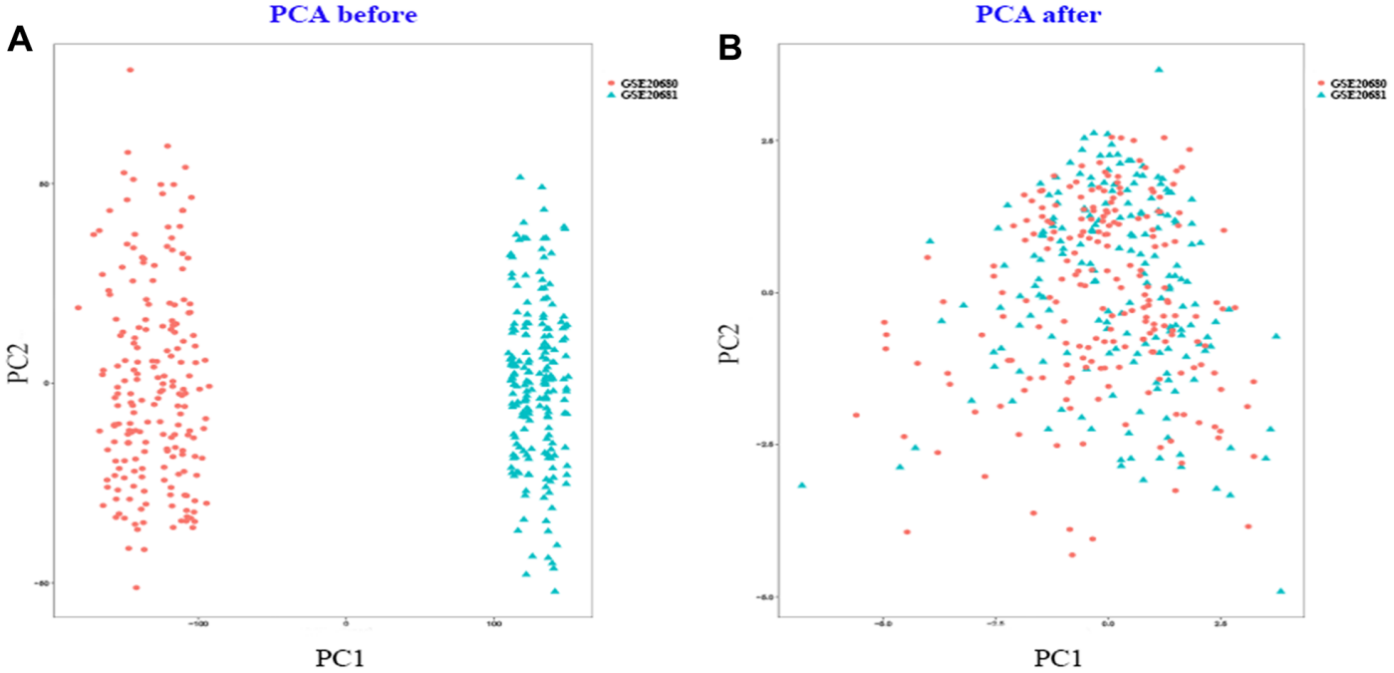
**PCA cluster plot.** (**A**) PCA cluster plot of GSE20680 and GSE20681 before sample correction and remove batch effect. (**B**) PCA cluster plot of GSE20680 and GSE20681 after sample correction and remove batch effect.

First of all, the batch effect between GSE20680 and GSE20681 was evaluated and visualized by PCA cluster diagram and the PCA results showed that there indeed existed batch effect between them (Figure 1A). Then these two gene expression matrices were normalized and processed by sva package in R. It was displayed using PCA cluster diagram after normalization and removing batch effect (Figure 1B). The results clearly indicated that the batch effect between GSE20680 and GSE20681 was successfully removed.

### WGCNA analysis in patients with CAD

**Figure 2:**
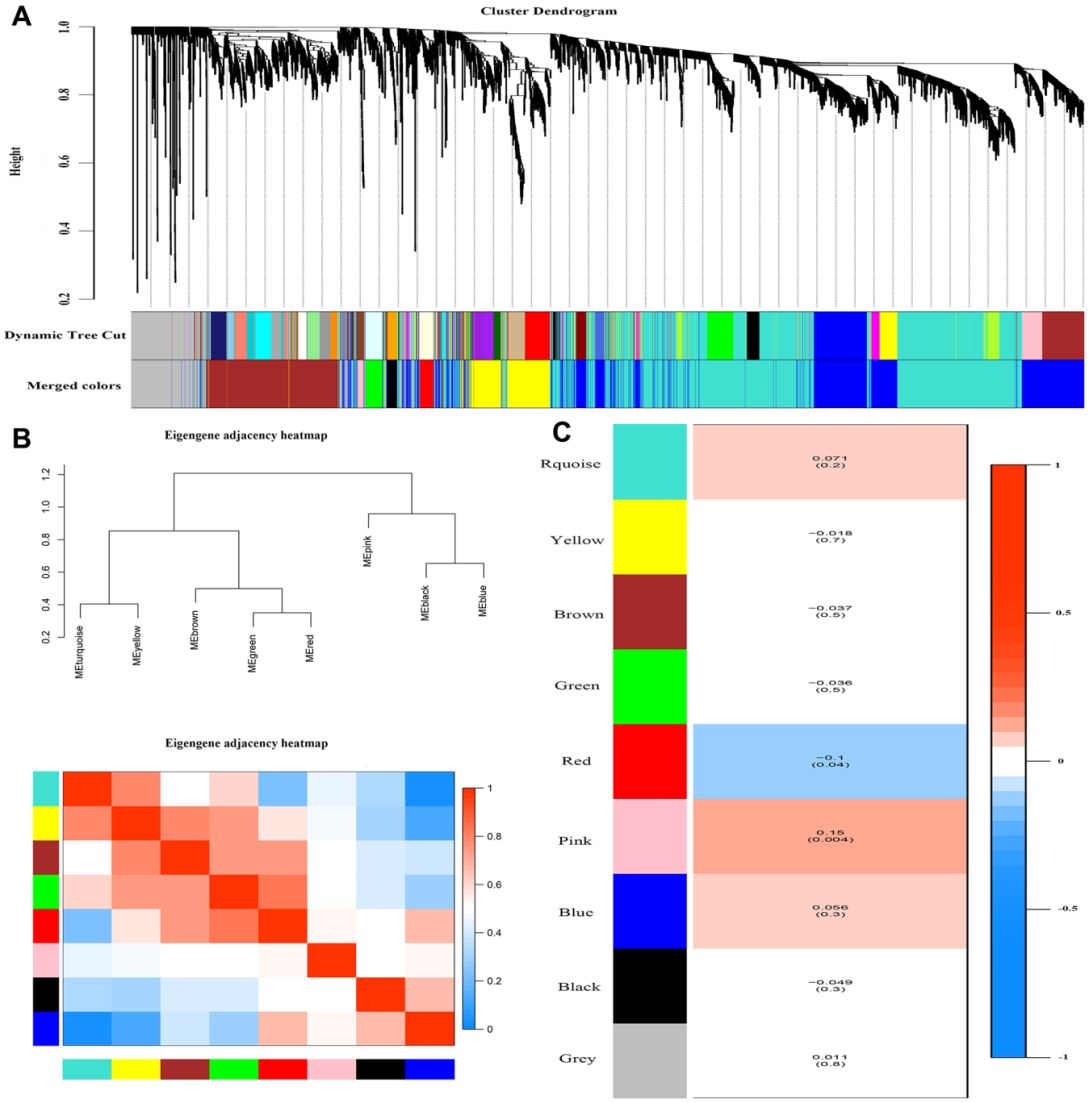
**Weighted correlation network analysis.** (**A**) Recognition module, each module was given an individual color as identifiers, including 8 different modules. (**B**) Co-expression similarity of entire modules based on hierarchical clustering of module eigengenes and the correlation between different modules, red indicates high adjacency (positive correlation) and blue low adjacency (negative correlation). (**C**) Correlation heat map of gene modules and phenotypes, the red is positively correlated with the phenotype; blue is negatively correlated with the phenotype.

WGCNA method was introduced to identify the key module using the expression profile with the top 25% highest variance. In summary, a total of 186 CAD samples and 207 control samples, with 4937genes expression profiles were included into the WGCNA analysis. We chose 11 as the β value to construct the scale-free network (Supplementary Figure 1A, Supplementary Figure 1B). 8 co-expression modules were identified and the number of genes in these modules ranged from 31 to 1954 (Figure 2A). The connectivity was calculated and cluster analysis was performed among the 8 modules (Figure 2B). To further analyze the association between the models and phenotype, we calculated the correlation coefficients of each model with CAD trait. The results showed that the pink module was positively and red module was negatively associated with CAD with statistical significance (r=0.15 and -0.1, respectively. Figure 2C).

### Functional enrichment analysis

**Figure 3:**
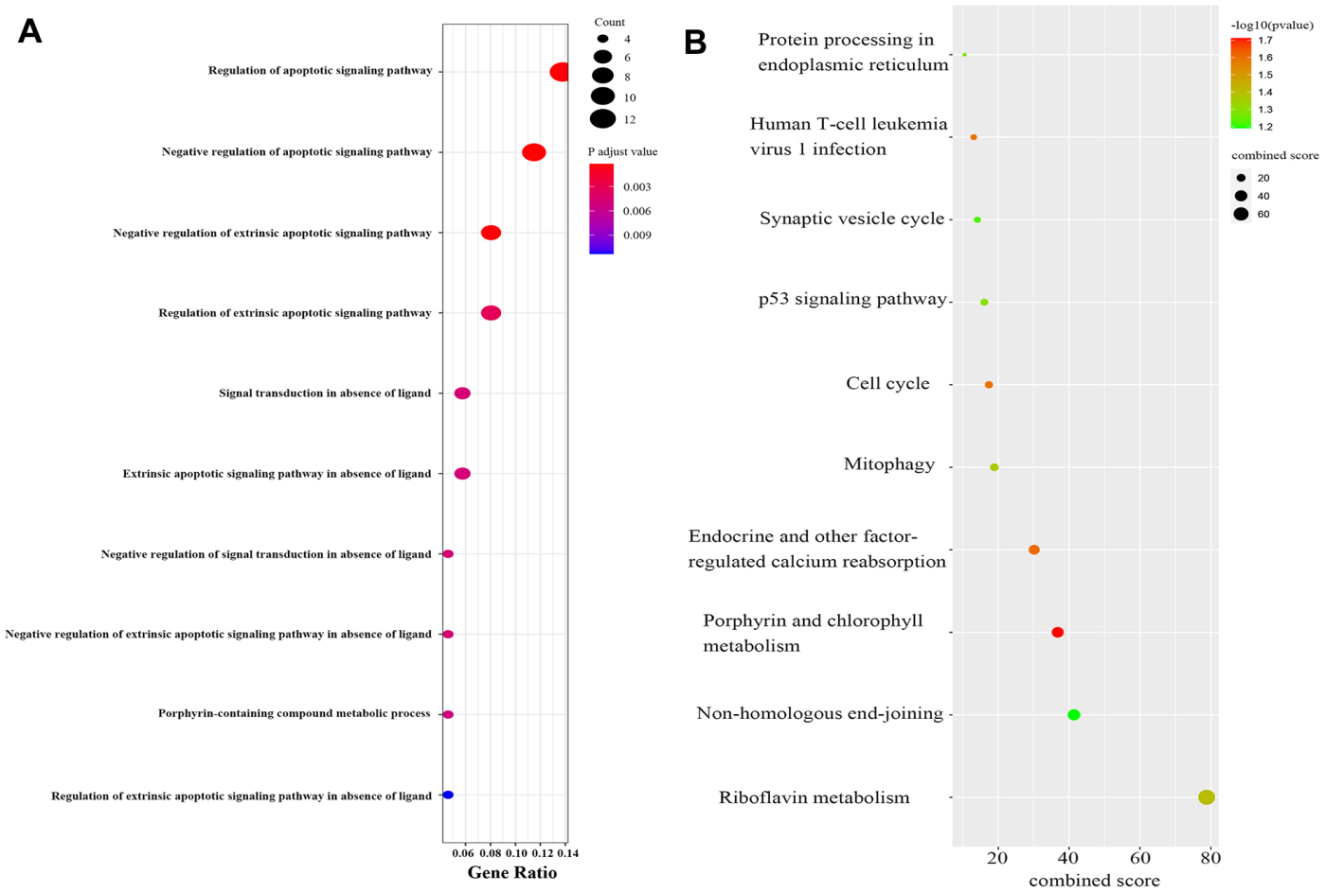
**Functional enrichment analysis of module genes.** (**A**) Biological processes of module genes, the significance of enrichment gradually increases from blue to red, and the size of the dots indicates the number of differential genes contained in the corresponding pathway. (**B**) KEGG pathways analysis of module genes. The significance of enrichment gradually increases from blue to red, and the size of the dots indicates the number of differential genes contained in the corresponding pathway.

Functional enrichment analysis was performed using the genes identified in red and pink modules. For biological pathway analysis, most enrichment terms were CAD-associated, including the regulation of apoptotic signaling pathway, negative regulation of apoptotic signaling pathway and extrinsic apoptotic signaling pathway, etc (Figure 3A). KEGG pathway analysis mapped module genes into riboflavin metabolism, Non-homologous end-joining, Porphyrin and chlorophyll, etc (Figure 3B). Taken together, we selected these two modules (with 104 genes contents) for further analysis in the next step.

### Screening and verification of diagnostic markers

**Figure 4:**
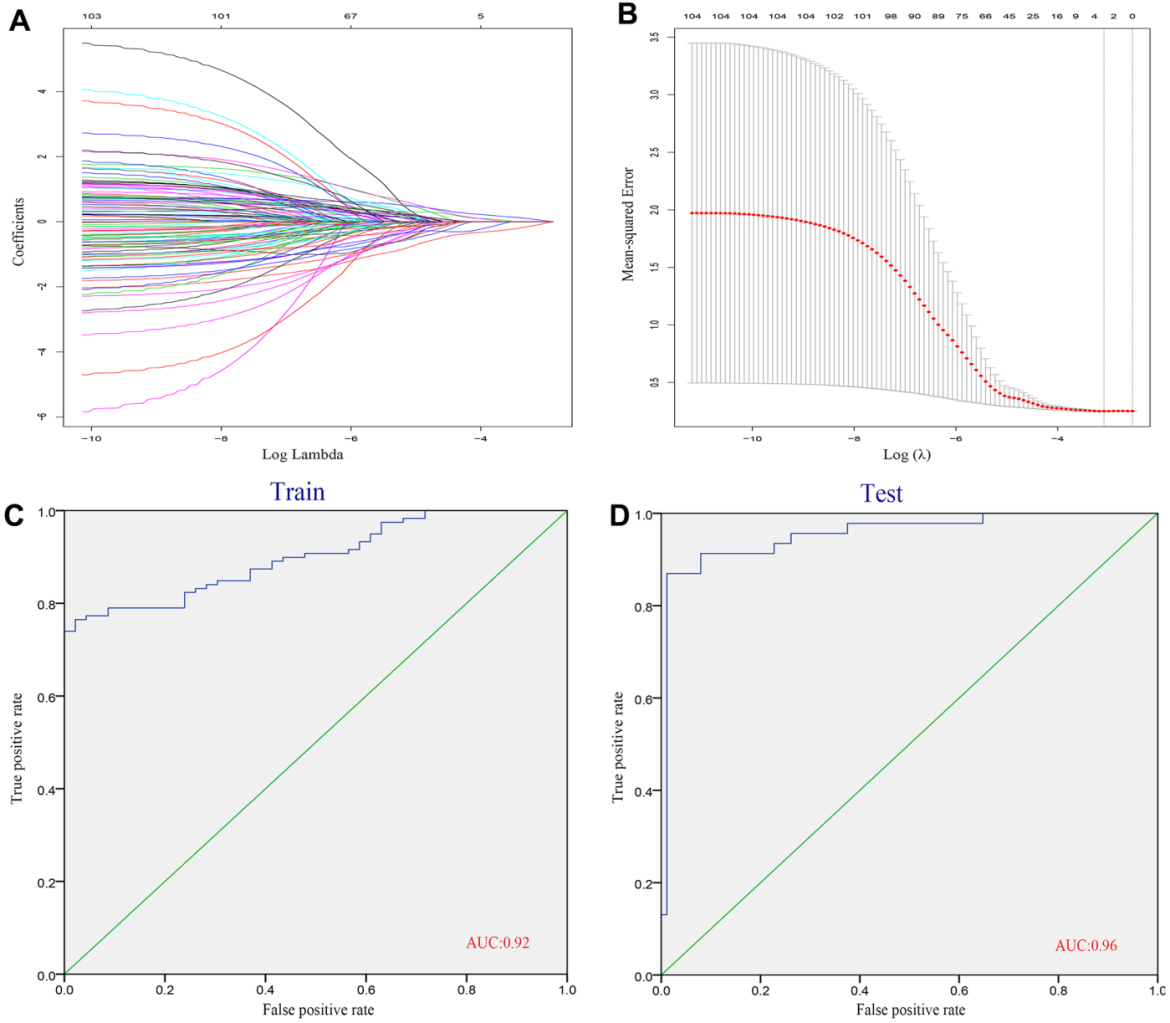
**Identification of diagnostic genes related to CAD.** (**A**, **B**) We conducted the LASSO method based on glmnet package and identified 3 diagnostic genes. (**C**) ROC curves analysis of train set. (**D**) ROC curves analysis of test set.

First, we extracted the genes of key modules obtained from WGCNA analysis to construct LASSO model. Three genes (ASCC2, LRRC18, SLC25A37) were identified using LASSO method and the value of lambda.min=0.04192 (Figure 4A, 4B). We constructed the formula using the expression level of these three genes and the coefficients obtained from LASSO: index=ASCC2*(-0.2382) + LRRC18*0.4071 + SLC25A37*0.2234. Next, we used the established model in the testing data sets to validate its performance. The diagnostic efficiency for training set was 0.92 and the AUC area reached 0.96 in testing set (Figure 4C, 4D), indicating that these biomarkers had high diagnostic value.

### External validation of the diagnostic markers in cohort study

**Figure 5:**
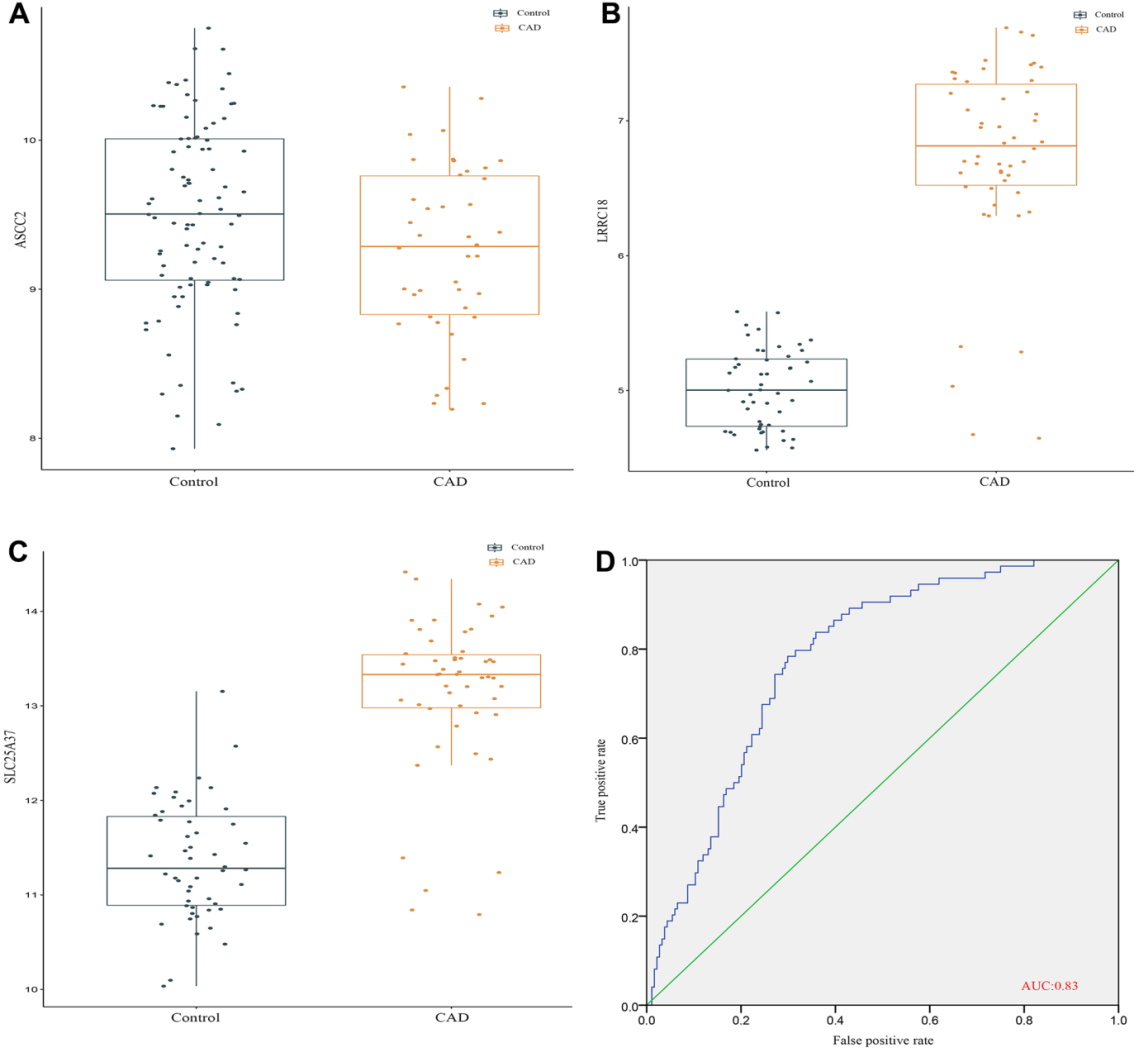
**External validation of the diagnostic markers in cohort study.** (**A**) The gene expression level of ASCC2. (**B**) The gene expression level of LRRC18. (**C**) The gene expression level of SLC25A37. (**D**) ROC curves analysis of validation set.

To further validate the effects of these biomarkers in CAD diagnosis, we first detected the expression of these genes in the peripheral blood sample from a cohort study in fourth affiliated hospital of China medical university. The results showed that the expression of ASCC2 displayed a negative trend between control group and CAD group (Figure 5A). In contrast, both of LRRC18 and SLC25A37 were highly expressed in CAD group than that in control group (Figure 5B, 5C). Furthermore, for the validation of these biomarkers in CAD diagnosis, we calculated the AUC area of these genes in the validation cohort and the value was 0.83 for prediction of CAD (Figure 5D).

### Immune cell infiltration results

**Figure 6:**
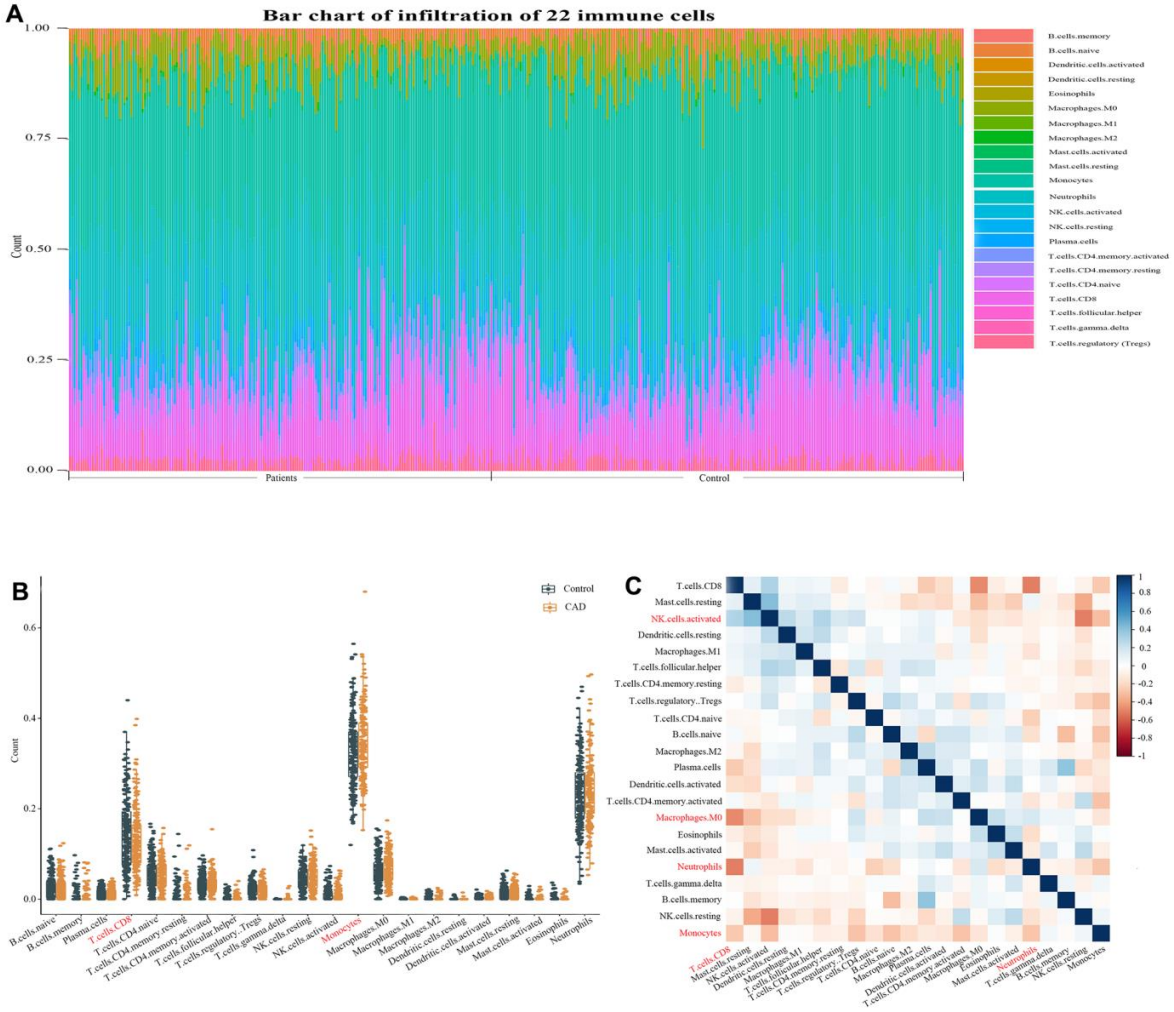
**Evaluation and visualization of immune cell infiltration.** (**A**) Immune cell types and ratios of CAD patients. (**B**) Boxplot diagram of the 22 types of immune cells. (**C**) Correlation heat map of 22 types of immune cells. The size of the colored squares represents the strength of the correlation; blue represents a positive correlation, red represents a negative correlation. The darker the color, the stronger the correlation.

To further research the differential expression of immune fractions between CAD patients and control group, we used the CIBERSORT algorithm to evaluate the association between CAD phenotype and immune cells infiltration. The relative proportion of immune cell subtypes was shown in the cumulative histogram (Figure 6A). The results showed that monocytes and CD8 T cells captured a obvious proportion, which indicated monocytes and CD8 T cells were vital in cardiovascular disease. Next, we calculated the number of these 22 immune cells in the sample, results suggested that the numbers of T cells CD8 (p=0.028) were lower and monocytes (p=0.028) were higher in the CAD patients in contrast with the control group (Figure 6B), respectively. However, the other 20 immune cells failed to display significant difference. Using a correlation matrix, we found that CD8 T cells had negative correlation with macrophages M0, neutrophils and monocytes, while it correlated positively with NK cells activated. We also found that monocytes had a negative correlation with neutrophils (Figure 6C).

### Correlation analysis between identified diagnostic markers and infiltrating immune cells

**Figure 7:**
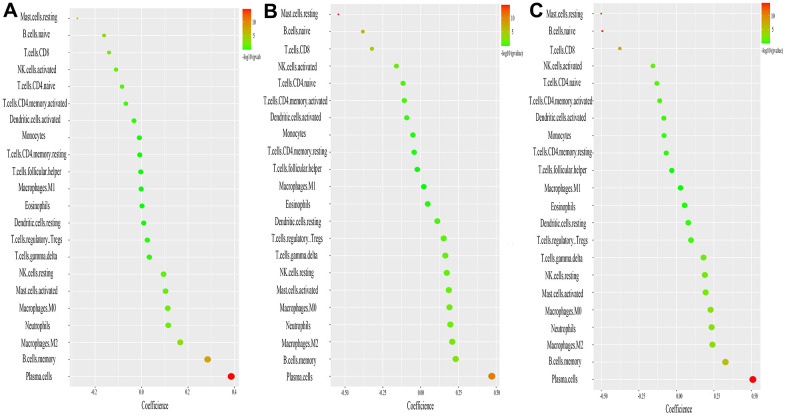
**Correlation between ASCC2, LRRC8, SLC25A37, and infiltrating immune cells.** (**A**) Correlation between ASC2 and infiltrating immune cells. (**B**) Correlation between LRRC18 and infiltrating immune cells. (**C**) Correlation between SLC25A37 and infiltrating immune cells. The size of the dots represents the strength of the correlation between genes and immune cells; the larger the dots, the stronger the correlation. The color of the dots represents the p-value, the greener the color, the lower the p-value. p < 0.05 was considered statistically significant.

The results displayed that ASCC2 had a positive correlation with resting plasma cells (r = 0.38, p = 0.000) and negative correlated with mast cells resting (r = −0.27, p = 0.000) and T cells CD8 (r= −0.13, p=0.000) (Figure 7A); LRRC18 demonstrated positive correlation with resting plasma cells (r =0.47, p = 0.000) and negative correlation with T cells CD8 (r= −0.33, p=0.000) and Mast cells resting (r= −0.54, p=0.000) (Figure 7B). SLC25A37 also demonstrated positive correlation with plasma cells (r=0.51, p=0.000) and negative correlation with T cells CD8 (r= −0.38, p=0.000) and Mast cells resting (r= −0.49, p=0.000) (Figure 7C).

## DISCUSSION

We downloaded the microarray expression profile between CAD patients and control sample from the GEO database and identified three genes (ASCC2, LRRC18 and SLC25A37) as key genes in CAD via integrated bioinformatics analysis. We identified red and pink modules in WGCNA analysis and found 104 genes in these two modules. We further used LASSO method to identify the key genes in differentiating CAD patients from normal people. We further studied the immune cells infiltration in CAD patients and found that several immune cells displayed differential expression between CAD patients and control group. Furthermore, we also found immune cells, including macrophages M0, mast cells resting and T cells CD8, had complicated association with ASCC2, LRRC18 and SLC25A37. Gene enrichment analysis indicated that these genes mainly enriched in apoptotic signaling pathway for biological pathway analysis, riboflavin metabolism for KEGG analysis.

In this paper we identified two modules that are correlated with CAD trait in WGCNA analysis. KEGG pathway analysis of these genes was mainly enriched in riboflavin metabolism. By reviewing the literature, we found riboflavin metabolism played important role in cardiovascular disease. The mechanism of riboflavin in cardiovascular disease mainly includes: riboflavin is an important coenzyme of flavin coenzyme, which is involved in the process of electron transfer in respiratory chain; riboflavin has the function of anti-platelet aggregation; it can also reduce lipid peroxidation by increasing superoxide dismutase and glutathione regeneration. It also promotes ischemic cardiomyocyte recovery via regulating sugar aerobic metabolism [12].

The proper response to DNA damage is associated with many pathological and physiological processes in human cells, including DNA replication, cell cycle and chromatin remodeling [13]. Activating Signal Cointegrator Complex (ASCC) may play a pivotal role during the transcriptional response among cell repair [13]. ASCC2, an important member of ASCC, could bind to the ribosome and protect cells from toxic effects [14]. However, there is no literature related to the relationship between ASCC2 and cardiovascular disease to our best knowledge. In our study, we found that the coefficient of ASCC2 in LASSO analysis is negative and the expression of ASCC2 in CAD patients is lower in contrast with the control group in cohort study. Furthermore, immune infiltration studies also indicated ASCC2 is associated with plasma cells and mast cells. Based on these evidences, we think it is reasonable to further study the role of ASCC2 in cardiovascular disease.

We also found that SLC25A37 had binding sites for RelB, which was the effector of NF-κB pathway [15]. SLC25A37 influences the gene expression of transport proteins in HUVECs response to TNFα stimulus. In addition, SLC25A37, as a member of SLC25 family, are widely expressed in the central nervous system and peripheral blood and may influence the function of mitochondrial [16]. Researchers also found that SLC25A37 regulated the proliferation and apoptosis in carcinoma through mitochondrion mediated iron channel [17]. Amigo implied that SLC25A37 was associated with heart development in zebra fish [18]. However, there is no study that is directly associated with cardiovascular disease in human.

Adverse innate immune responses have been implicated in several disease processes. Monocytes subsets are key in atherogenesis and the inflammatory cascade in cardiovascular disease. Upregulated activity and counts of monocyte are associated with clinical indices of atherosclerosis, heart failure syndromes and CKD [10]. T lymphocytes are important immune cells *in vivo* and can be divided into CD4, CD8 cell subsets according to their surface markers and functions. CD8 T cells have dual effects in atherosclerosis. Previous studies have shown that CD8 T cells could secret many inflammatory cytokines exacerbating inflammatory responses and increasing instability of atherosclerotic plaque [19], On the contrary, cytotoxic activity targeting antigen presenting cells and regulatory CD8 T-cell subsets could inhibit atherosclerosis by alleviating immunity reaction [19]. Other immune cell types, including master cells [20] and neutrophil cells [21], also play pivotal role in the development of cardiovascular disease. To further evaluate the types and proportions of immune cells in CAD, the CIBERSORT website was used to perform a comprehensive evaluation of 22 types of immune cells infiltration in CAD patients. The results displayed a decreased infiltration of CD8 T cells while an increased infiltration of monocytes, which were similar to the previous studies [22, 23]. In contrast, the persistent T-cell response triggered by myocardial infarction is associated with subsequent continuous left ventricular remodeling resulting in heart failure and cardiac arrest [24], indicating that the immune system is very complex in the occurrence and development of CAD. As previous discussed, CD8 T cells had pleural effects on atherosclerosis and our research indicated the expression level of CD8 T cells is lower than that in the control group. This meant that CD8 T cells could inhibit atherosclerosis in the GEO datasets used in this paper. However, it is still unclear whether the number of CD8 T cells in peripheral blood sample could reflect its infiltration condition in the vessel wall [25]. Besides, this paper revealed the interaction of 22 types of infiltrated immune cells in CAD. CD8 T cells negatively correlated with Macrophages M0, Neutrophils and monocytes. The immune cells infiltration analysis implied that there may be a complicated network in cardiovascular disease. CD8 T cells maybe an important node in the network of immune regulation network in atherosclerosis. However, the underlying mechanisms of these correlations between infiltrated immune cells need *in vivo* and *in vitro* experiments to validate. We also found that macrophages M0, mast cells resting, and T cells CD8 have complicated association with ASCC2, LRRC18 and SLC25A37. The association of these genes with immune system is still unclear. It will need more research to study the underlying mechanism of these genes in immune system in atherosclerotic disease.

In summary, we identified ASCC2, LRRC18, and SLC25A37 as diagnostic markers for CAD. This paper also indicated CD8 T cells and monocytes maybe related with the initiation and progression of CAD. Besides, ASCC2, LRRC18 and SLC25A37 had negative correlation with CD8 T cells. The mechanism between immune cells and these key genes may be of great importance in the onset and progression of CAD and related research of these genes could provide new therapeutic insight for cardiovascular disease.

## MATERIALS AND METHODS

### Download GEO database and data preprocessing

First, we researched with (“coronary artery disease” or “CAD”) and (“whole blood” or “peripheral blood”) in GEO database to find the interested expression data. After excluding irrelevant datasets, GSE20680 and GSE20681 were successfully downloaded. The GSE20680 contains 195 samples which including 87 CAD patients with vessel stenosis >50% and 108 control samples. The GSE20681 contains 198 samples which including 99 CAD patients with vessel stenosis >50% and 99 control samples. These two datasets were based on GPL4133. The peripheral blood gene expression profile was annotated using the GPL4133 platform data, and then quantile normalization was performed with preprocessCore R package. When multiple probe sets were annotated by the same gene, the maximum intensity was selected. Totally, the expression profile contained 19749 genes for further study. Batch effect was removed using ComBat function [26].

### WGCNA construction

Out of 19,749 genes, the top 25% genes (4937 genes) with high expression variance were selected to be analyzed by WGCNA method. In WGCNA analysis, the Pearson correlation coefficient between any two genes among the 4937 genes was calculated. Then adjacency matrix was constructed with the proper β value when the R^2^ was first over 0.8 [27]. Following that, topological overlap matrix was generated using blockwiseModules function in R software with the parameters as follows: minModuleSize = 30; power = 11 and mergeCutHeight = 0.25 [28]. Then, cluster dendrogram was constructed and visualized by plotDendroAndColors function and the modules obtained by merging the branches of the clustering tree into different gene modules with dynamic tree cut algorithm were visualized by different colors [28].

### Module-trait correlation and functional enrichment analysis

The PCA assay was used to find the eigengene for each identified module [29]. Module-trait correlations analysis was performed using multivariable linear regression method based on the eigengene. Gene modules significantly related to CAD were selected and functional analyzed using GO and KEGG database. The clusterProfiler package in R software was used to carried out the GO and KEGG analysis [30].

### Identification and validation of diagnostic markers

LASSO regression method [31] was introduced to select the key genes for establishing diagnostic model for CAD. The “glmnet” package was used in LASSO regression analysis [32]. The expression matrixes of the GSE20680, GSE20681 datasets were merged after data preprocessing. The merged independent dataset was randomly divided into training and testing dataset by 2:1 ratio. Then the diagnostic accuracy of the obtained diagnostic markers in training and testing dataset was achieved, respectively.

### Diagnostic value of identified genes in validation cohort

Validation cohort was randomly recruited from the patients admitted to Fourth affiliated hospital of China Medical University between May 2016 and December 2017 for suspected unstable angina who complained with chest pain. All the subjects in the validation cohort received coronary angiography. The validation cohort included 100 patients with vessel stenosis >50% and 100 patients with vessel stenosis <50%. The criterion of suspected CAD: patients complained with chest pain without cardiovascular disease history. The research protocol was approved by the institution of the author and all the subjects in the study signed informed consents. The gene expression level of identified key genes in the validation cohort was measured by PCR method. The protocol of PCR was supplied in supplementary file.

### Evaluation of immune cell infiltration and its correlation with key genes

We performed immune infiltration by using CIBERSORT.R script downloaded from CIBERSORT website [11]. After obtaining the expression matrix of immune cell according to the instruction of the CIBERSORT website, we used “ggplot2” package to draw the cumulative histogram to visualize the proportion of 22 immune cells infiltration in CAD patients. We also use “ggplot2” package to draw boxplot diagrams to display the expression difference of 22 types of infiltrating immune cells. The Pearson correlation coefficient between each immune cell was calculated and the results were visualized by correlation heatmap using “corrplot” package in R software. The Spearman correlation coefficient between identified key genes with infiltrating immune cells was calculated with “ggstatsplot” package and then visualized by “ggplot2” package.

### Statistical analysis

All the data processing and statistical analysis was performed in R software (version 3.6.0). The value of area under the receiver-operator characteristics (ROC) curve (AUC) was used to determine the accuracy of the newly established model. The calibration performance was evaluated using calibration curves and Hosmer-Lemeshow test. The *p* value below 0.05 at two-sided was considered statistical significance.

### Data availability statement

The data that support the findings of this study are available from the corresponding author upon reasonable request.

## Supplementary Material

Supplementary Methods

Supplementary Figure 1
