# Supplementary material for: Identification of key genes in coronary artery disease: an integrative approach based on weighted gene co-expression network analysis and their correlation with immune infiltration
Source: Aging (Albany NY). 2021 Mar 3;13(6):8306–19. doi: 10.18632/aging.202638 (PMC8034924; doi:10.18632/aging.202638)
Supplement: Supplementary Methods [file aging-13-202638-s001.pdf]

SUPPLEMENTARY METHODS

PCR protocol

mRNA expression was evaluated using RT- PCR. Total RNA was extracted from plasma using TRIzol solution (Invitrogen, Carlsbad, USA) following the instructions of manufacturer. Then, nanodrop ND- 1000 (Biotek, Vermont, USA) was used to detect the concentration and purity of total RNA, and the integrity of total RNA was detected using agarose gel electrophoresis. Finally, 1 μg of total RNA with A260/A280 value of 1.9-2.1, A260/A230 >2.0 was used for cDNA synthesis using transcription kit (TOYOBO, Osaka, Japan). Subsequently, the SYBR Premix EX Taq kit (KAPA, Boston, USA) was used to evaluate the expressions using GAPDH as an internal reference. The relative quantity of mRNAs was calculated by the $2^{-\Delta\Delta Ct}$ formula. The cDNA was amplified in triplicate (3 technical replicates) per reference gene and biological replicate (sample).

Primer sequence

GAPDH:	F: CATACCAGGAAATGAGCTTG	
	R: ATGACATCAAGAAGGTGGTG	
ASCC2:	F: TTGGTGAGGGCTTCGCCCT	
	R: AGGGCGAAGCCCTCACCAA	
LRRC18:	F: AAGGACCTGGCGGCCGACC	
	R: GTAGTACTCCGCGCAGGCC	
SLC25A37:	F: GATGGGGACAGCCGAGATG	
	R: ACCGGGTACATGACCGAGT	

Module gene

Red module

ADIPOR1	AK1	ANKH	ANKRD9	AP2A1	AP2M1
ASCC2	BCL2L1	BLVRB	BSG	C22orf13	C2orf24
CDC34	CSDA	DNAJB2	DPM2	EIF2AK1	EPB49
FAM100A	FAM117A	FIS1	FKBP8	FZR1	GATA1
GMPR	GPR146	GRINA	GYPC	H2AFJ	HAGH
HDGF	HMBS	HPS1	HSPB1	KIAA1539	KLF1
LOC442211	LYL1	MAF1	MARK3	MCOLN1	MXD4
OR2W3	OSBP2	PHOSPHO1	PNPLA2	POLL	POLR1D
PRDX2	RAD23A	RANBP10	RILP	RNF10	RNF123
RNF187	RUNDC3A	SFRP2	SH3GL1	SIAH2	SLC1A5
SLC25A37	SLC25A39	SLC38A5	SLC6A10P	SLC6A8	SPTB
ST6GALNAC4	TCF3	TFDP1	TMCC2	TSTA3	WDR45
ZER1					

Pink module

ABHD3	ATPBD4	C17orf71	C5orf42	C7orf11	C9orf130
CHEK2	CXorf36	DOK5	GLT8D3	GRAMD2	HEPACAM2
ITGAV	KCNE2	KIAA1549	LCE2C	LIN28	LOC51152
LOC650157	LOC652494	LRRC18	NAPG	NPAL2	PAK6
PHKA1	RANGAP1	TERT	TTC23	U2AF1L4	ZNF296
ZNF596					
