# Supplementary material for: Identification of key genes in coronary artery disease: an integrative approach based on weighted gene co-expression network analysis and their correlation with immune infiltration
Source: Aging (Albany NY). 2021 Mar 3;13(6):8306–19. doi: 10.18632/aging.202638 (PMC8034924; doi:10.18632/aging.202638)
Supplement: Supplementary Figure 1 [file aging-13-202638-s002.pdf]

Supplementary Figure

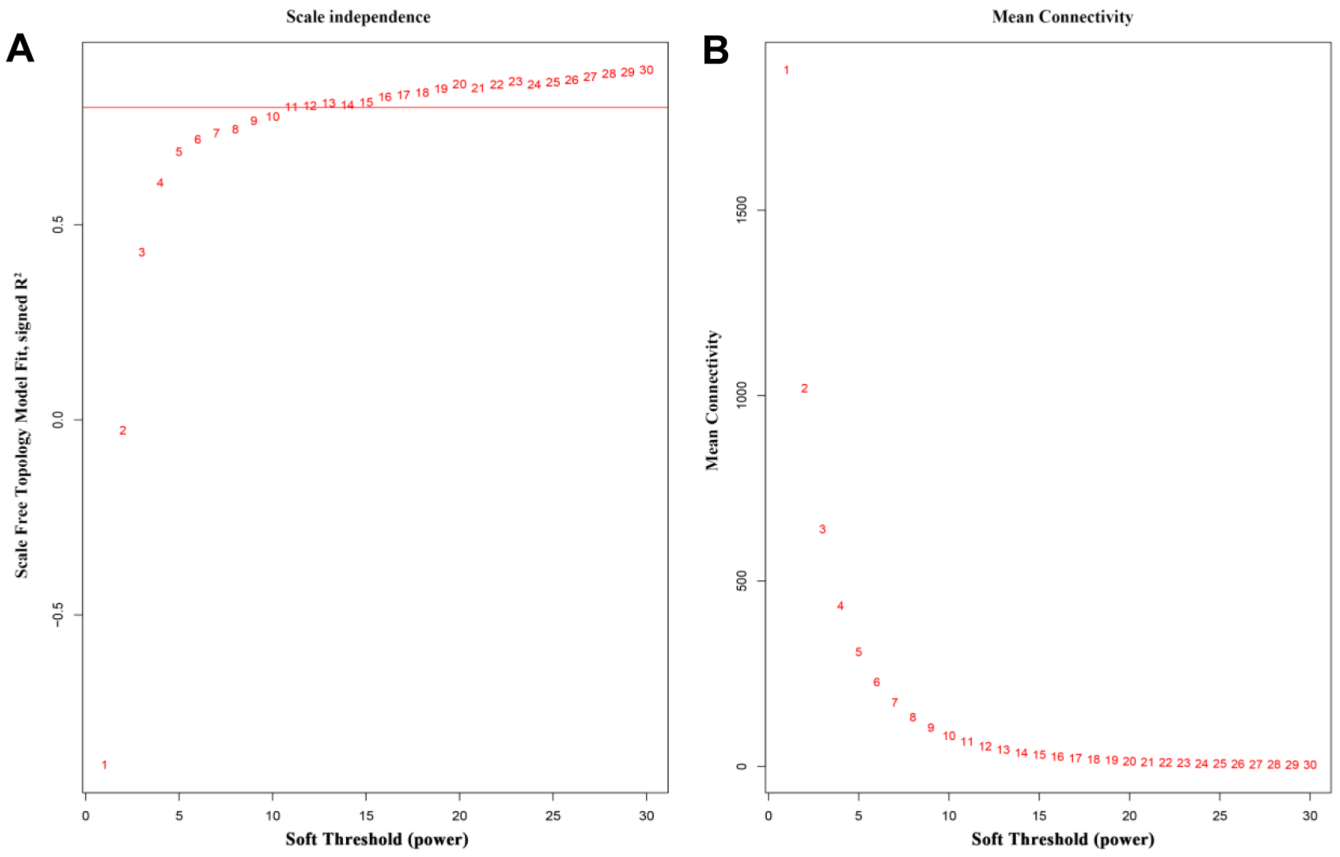

Supplementary Figure 1. The soft threshold of WGCNA. (**A**) Analysis of the scale-free fit index for various soft thresholding powers. (**B**) Analysis of the mean connectivity for various soft-thresholding powers.
